# Efficacy of Artificial Tears Based on an Extract of *Artemia salina* Containing Dinucleotides in a Rabbit Dry Eye Model

**DOI:** 10.3390/ijms222111999

**Published:** 2021-11-05

**Authors:** Carlos Carpena-Torres, Jesus Pintor, Fernando Huete-Toral, Alba Martin-Gil, Candela Rodríguez-Pomar, Alejandro Martínez-Águila, Gonzalo Carracedo

**Affiliations:** 1 Ocupharm Research Group, Department of Optometry and Vision, Faculty of Optics and Optometry, Complutense University of Madrid, C/Arcos de Jalón 118, 28037 Madrid, Spain; ccarpena@ucm.es (C.C.-T.); amarting@ucm.es (A.M.-G.); candelarodriguezpomar@gmail.com (C.R.-P.); 2 Ocupharm Research Group, Department of Biochemistry and Molecular Biology, Faculty of Optics and Optometry, Complutense University of Madrid, C/Arcos de Jalón 118, 28037 Madrid, Spain; jpintor@ucm.es (J.P.); fhueteto@ucm.es (F.H.-T.); alejandromaguila@gmail.com (A.M.-Á.)

**Keywords:** dry eye, *Artemia salina*, dinucleotides, secretagogue, purinergic signaling

## Abstract

(1) Background: *Artemia salina* is a brine shrimp containing high concentrations of dinucleotides, molecules with properties for dry eye treatment. For this reason, the purpose of the study was to evaluate the effect of the artificial tears based on an extract of *Artemia salina* in a rabbit dry eye model. (2) Methods: A prospective and randomized study was carried out. Twenty rabbits were divided into 4 groups (*n* = 5, each group): healthy rabbits, dry eye rabbits, dry eye rabbits treated with hypromellose (HPMC), and dry eye rabbits treated with *Artemia salina*. Dry eye was induced by the topical instillation of 0.2% benzalkonium chloride. The measurements were performed before and after the treatment for 5 consecutive days. (3) Results: The topical instillation of artificial tears containing *Artemia salina* showed beneficial effects on tear secretion, tear break-up time, corneal staining, the density of Goblet cells, heigh of mucin cloud secreted by these cells, and mRNA levels of IL-1β and MMP9 in conjunctival cells. Compared with the HPMC, there was a statistically significant improvement (*p* < 0.05) with the *Artemia salina* in all the variables under study, except for the conjunctival hyperemia, density of Goblet cells, and mRNA levels of IL-6. (4) Conclusions: The potential of artificial tears based on *Artemia salina* as a secretagogue agent for dry eye treatment was confirmed, opening the door for future clinical trials and studies to extrapolate the findings for dry eye patients.

## 1. Introduction

Dry eye was defined by the Tear Film and Ocular Surface Society (TFOS) as “a multifactorial disease of the ocular surface characterized by a loss of homeostasis of the tear film, and accompanied by ocular symptoms, in which tear film instability and hyperosmolarity, ocular surface inflammation and damage and neurosensory abnormalities play etiological roles” [1]. This condition affects a large portion of the world’s population, reaching a prevalence of 50% in some regions of the planet [2]. For the treatment of dry eye, a remarkable family of compounds depicting interesting physiological properties on the ocular surface is dinucleoside polyphosphates, also known as dinucleotides [3].

Dinucleoside polyphosphates are formed by two nucleotides linked by a variable number of phosphates (from 2 to 7) and play an important role in the diagnosis and treatment of dry eye [4]. These compounds activate cell surface purinergic P2 receptors, both P2X ionotropic [5] and P2Y metabotropic [6] receptors. P2 receptors have been found in different parts of the eye such as the ocular surface, anterior and posterior chambers, and retina [7]. The activation of P2Y_2_ receptor, a subtype of P2Y receptors, produces chloride efflux and water movement into the extracellular medium [4,8,9].

On the ocular surface, P2Y_2_ receptor has been described in the cornea, conjunctiva, and lacrimal gland [10]. The activation of P2Y_2_ receptor via topical instillation of diadenosine tetraphosphate (Ap_4_A) and diuridine tetraphosphate (Up_4_U) demonstrated its capability to stimulate aqueous [11,12,13,14], mucinous [15], and lipidic [16] components of tears, as well as to accelerate corneal re-epithelization [15,17,18]. Additionally, the instillation of these compounds increases the corneal permeability [19,20] and the levels of both lysozyme [21] and lactoferrin [22] in tears.

From a clinical viewpoint, the scientific evidence supports the safety and security of the commercial form of Up_4_U (Diquafosol®) as a secretagogue agent for the treatment of dry eye [23,24]. The Diquafosol®, which is only commercially available in Asia, demonstrated its efficacy to improve the signs and symptoms of dry eye in patients with Sjögren syndrome [25], aqueous deficient dry eye [26], evaporative dry eye [27], dry eye associated with cataract surgery [23], contact lens wearers [28], and even in healthy subjects [29].

Recently, our research group developed artificial tears based on an extract of 4% *Artemia salina* [30], a brine shrimp containing high concentrations of dinucleotides, mainly diguanosine tetraphosphate (Gp_4_G), but also others such as diguanosine triphosphate (Gp_3_G) and diphosphate (Gp_2_G), or guanosine-adenosine tetraphosphate (Gp_4_A) [31,32]. The concentration of 4% *Artemia salina* was chosen in a previous study where the effect of short-term instillation of different concentrations of the brine shrimp (2%, 4%, 6%, 8%, and 10%) was evaluated in healthy rabbits [30]. The 4% *Artemia salina* manifested the best results in increasing tear secretion and improving corneal epithelial damage, also confirming its safety over the ocular surface.

The purpose of the current study was to evaluate the effect of the artificial tears based on an extract of 4% *Artemia salina* on lacrimal function and ocular surface damage and inflammation in a rabbit dry eye model. The dry eye model was induced by topical instillation of 0.2% benzalkonium chloride (BAC) for 5 consecutive days, a method previously validated [33], while the treatment with the artificial tears was applied simultaneously.

## 2. Results

Table 1 summarizes the values of all the variables under study before (PRE) and after (POST) the instillation of the different treatments for 5 consecutive days and the statistical comparison between PRE and POST measurements.

### 2.1. Tear Secretion and Tear Break-Up Time

**Figure 1:**
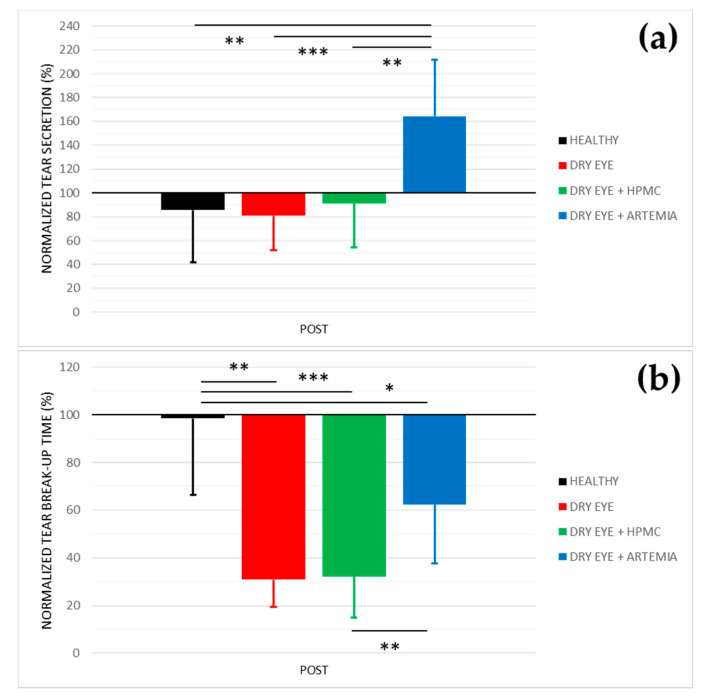
Normalized effect on tear secretion (**a**) and tear break−up time (**b**) of the instillation of the different treatments for 5 consecutive days. The values higher or lower than 100% represent an increase or decrease in comparison with their baseline, respectively. The statistical comparison was performed between the different groups (*n* = 5, each group). * *p* < 0.05, ** *p* < 0.01, *** *p* < 0.001, Student’s *t*−test for independent samples.

Figure 1 shows the normalized effect after the instillation of the different treatments on both tear secretion and tear break-up time, and the statistical comparison between all the groups.

Concerning tear secretion, the group of rabbits with dry eye treated with *Artemia salina* was the only one that showed a statistically significant increase of 64.38 ± 18.41% in comparison with the rest of the groups (*p* < 0.05). The other groups did not show statistical differences between them nor changes in comparison with their baseline measurements (*p* ≥ 0.05).

In relation to tear break-up time, there was a statistically significant deterioration in the three groups of rabbits with induced dry eye in comparison with the healthy rabbits (*p* < 0.05). This deterioration was 69.13 ± 25.31% in the dry eye group, 67.78 ± 36.16% in the dry eye + HPMC group, and 37.73 ± 14.85% in the dry eye + *Artemia salina* group. Additionally, the treatment with *Artemia salina* in the rabbits with induced dry eye statistically improved the tear break-up time in comparison with the instillation of HPMC (*p* = 0.001).

### 2.2. Slit-Lamp Examination

**Figure 2:**
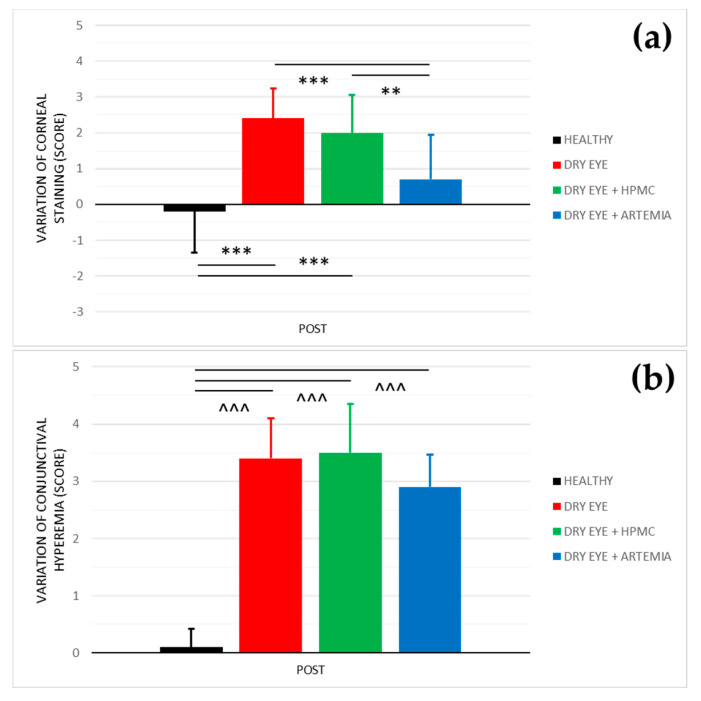
Variation of corneal staining (**a**) and conjunctival hyperemia (**b**) after the instillation of the different treatments for 5 consecutive days. The values higher or lower than 0 represent a deterioration or an improvement in comparison with their baseline, respectively. The statistical comparison was performed between the different groups (*n* = 5, each group). ** *p* < 0.01, *** *p* < 0.001, Student’s *t*-test for independent samples (normal distributions). ^^^ *p* < 0.001, Mann–Whitney U test (non-normal distributions).

Figure 2 shows the effect after the instillation of the different treatments on both corneal staining and conjunctival hyperemia, and the statistical comparison between all the groups. The effect on both variables was not normalized in percentage because they are discrete variables.

Regarding corneal staining, both healthy rabbits and the rabbits with induced dry eye treated with *Artemia salina* showed a statistically significant improvement in comparison with the rabbits with dry eye not treated and treated with HPMC (*p* < 0.05). On the other hand, despite the rabbits with dry eye being treated with *Artemia salina*, presenting a deterioration in comparison with their baseline, there were no statistical differences with the healthy rabbits (*p* = 0.109).

In terms of conjunctival hyperemia, there was a statistically significant deterioration in the three groups of rabbits with induced dry eye in comparison with the healthy rabbits (*p* < 0.05). Furthermore, the treatments with *Artemia salina* and HPMC had no effect since they showed no statistical differences with the rabbits with dry eye used as positive controls (*p* ≥ 0.05).

### 2.3. Conjunctival Cytology

**Figure 3:**
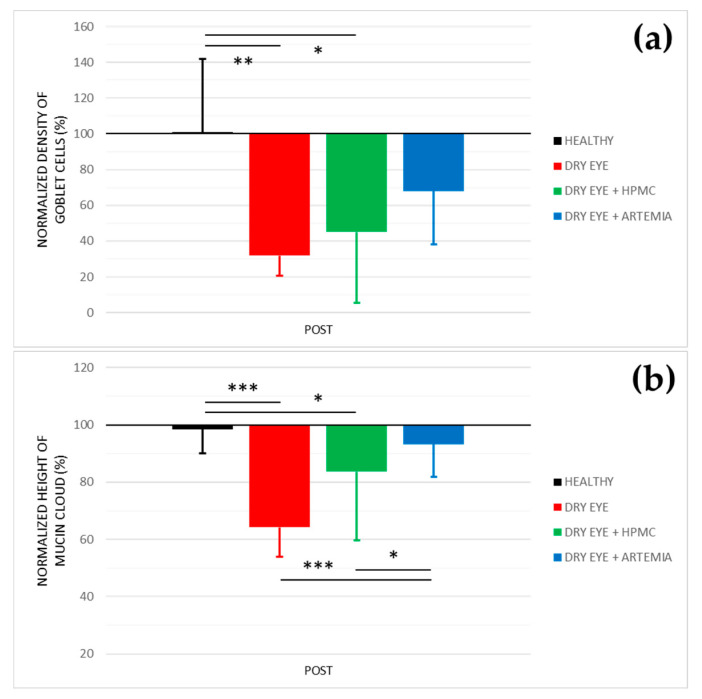
Normalized effect on the density of Goblet cells (**a**) and height of mucin cloud (**b**) of the instillation of the different treatments for 5 consecutive days. The values higher or lower than 100% represent an increase or decrease in comparison with their baseline, respectively. The statistical comparison was performed between the different groups (*n* = 5, each group). * *p* < 0.05, ** *p* < 0.01, *** *p* < 0.001, Student’s *t*-test for independent samples.

**Figure 4:**
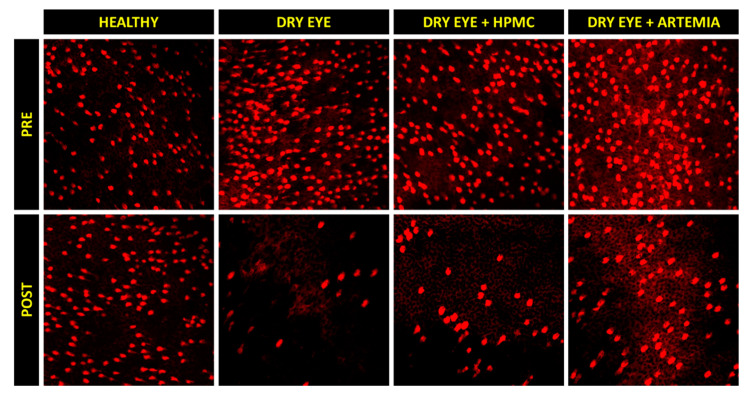
Representative images of the density of Goblet cells before (PRE) and after (POST) the instillation of the different treatments for 5 consecutive days. A decrease can be observed in the density of Goblet cells (brightest cells) in the three groups of rabbits with induced dry eye, but this decrease occurred in a lesser magnitude with the artificial tears based on *Artemia salina*.

Figure 3 shows the normalized effect after the instillation of the different treatments on both density of Goblet cells and height of mucin cloud, and the statistical comparison between all the groups, while Figure 4 shows representative images used to quantify the density of Goblet cells in the different groups.

Concerning the density of Goblet cells, the two groups of rabbits with induced dry eye treated or not treated with HPMC suffered a statistically significant deterioration of 68.03 ± 24.10% and 55.00 ± 48.29%, respectively, in comparison with the healthy rabbits (*p* < 0.05). Conversely, despite the rabbits with dry eye being treated with *Artemia salina* they also showed a deterioration compared with the healthy rabbits, these differences, however, were not statistically significant (*p* = 0.117).

Regarding the height of mucin cloud, again the two groups of rabbits with induced dry eye treated or not treated with HPMC suffered a deterioration of 35.81 ± 5.78% and 16.31 ± 4.70%, respectively, which was statistically significant in comparison with the healthy rabbits and the rabbits with dry eye treated with *Artemia salina* (*p* < 0.05). In addition, the treatment with *Artemia salina* in the rabbits with dry eye showed similar results to the healthy rabbits (*p* = 0.329).

### 2.4. Quantitative PCR

**Figure 5:**
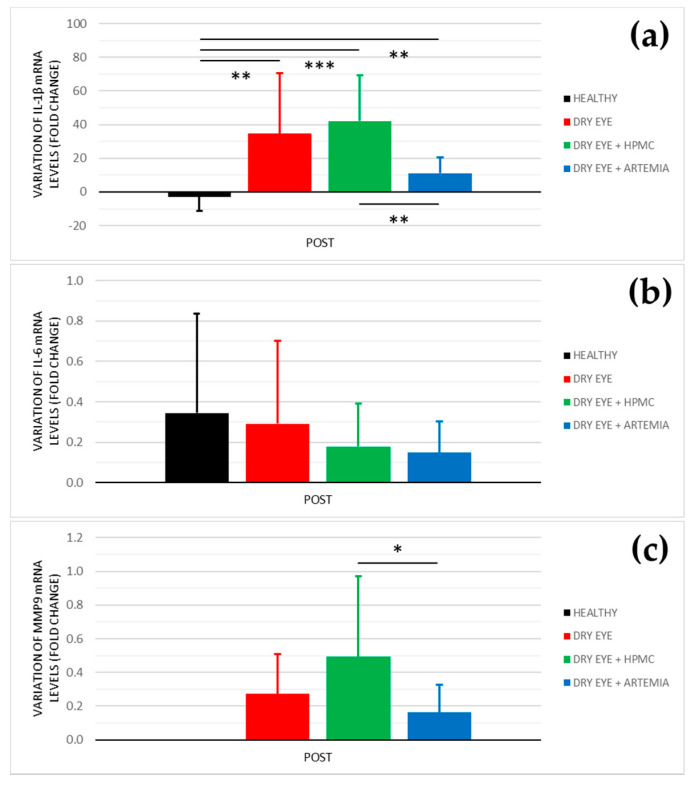
Variation of mRNA levels of IL−1β (**a**), IL−6 (**b**), and MMP9 (**c**) after the instillation of the different treatments for 5 consecutive days. The values higher or lower than 0 represent a deterioration or an improvement in comparison with their baseline, respectively. The statistical comparison was performed between the different groups (*n* = 5, each group). * *p* < 0.05, ** *p* < 0.01, *** *p* < 0.001, Mann–Whitney U test.

Figure 5 shows the effect after the instillation of the different treatments on the mRNA levels of IL-1β, IL-6, and MMP9, and the statistical comparison between all the groups. Previously, these results were normalized with the signal of the HPRT1 gene (Table 1), the reason why they were not normalized again in percentage.

In the levels of IL-1β, the three groups of rabbits with induced dry eye showed a fold increase of 34.611 ± 35.999 in the dry eye group, 42.014 ± 27.068 in the dry eye + HPMC group, and 11.114 ± 9.537 in the dry eye + *Artemia salina* group that was statistically significant in comparison with the healthy rabbits (*p* < 0.05). Additionally, the treatment with *Artemia salina* produced a decrease in the mRNA expression of IL-1β in comparison with the HPMC (*p* = 0.001).

Regarding the levels of IL-6, there were no statistically significant differences between all the groups (*p* ≥ 0.05), despite the rabbits with dry eye treated with *Artemia salina* and HPMC showed lower values.

In relation to the levels of MMP9, the quantitative PCR did not detect signals for the POST measurements of the healthy rabbits. However, in the rabbit with induced dry eye, the treatment with *Artemia salina* decreased the mRNA expression of MMP9 in comparison with the HPMC (*p* = 0.021). The fold change was 0.162 ± 0.165 with the *Artemia salina* and 0.494 ± 0.477 with the HPMC.

## 3. Discussion

The current study reported on the effect of artificial tears based on an extract of 4% *Artemia salina* in a rabbit dry eye model. The treatment with these artificial tears manifested beneficial effects on tear secretion, tear break-up time, corneal staining, the density of Goblet cells, height of mucin cloud, and inflammatory biomarkers. Additionally, the safety of these artificial tears was already confirmed in a previous study performed in healthy rabbits by our research group [30].

In terms of tear secretion, the topical instillation of artificial tears with 4% *Artemia salina* produced an increase of 64% in the rabbits with induced dry eye (see Figure 1), values slightly higher than those previously reported by our research group after the instillation of the same extract in health rabbits (44%) [30], but both studies showed an increase of around 4–5 μL compared with their baseline. The secretagogue effect on the aqueous component of tears was associated with the agonist action of dinucleoside polyphosphates present in *Artemia salina*, especially Gp_4_G, on P2Y_2_ receptors expressed in the conjunctival epithelium [11,12,13,14]. Nevertheless, it is still unknown whether Gp_4_G or other nucleotides present in the crustacean specifically activate the P2Y_2_ receptor as other dinucleotides such as Ap_4_A and Up_4_U. In this regard, different studies found that the topical instillation of Ap_4_A increased tear secretion by around 40–50% in healthy New Zealand white rabbits through the activation of the P2Y_2_ receptor [11,13]. The topical instillation of Up_4_U or Diquafosol® also demonstrated its capability to stimulate tear secretion by 33% in the same rabbits [12,14].

On the other hand, it should be noted that the instillation of 0.2% BAC to induce the dry eye did not decrease tear secretion in comparison with the group of healthy rabbits (see Figure 1), contrary to what happened in previous experiments [33]. This fact would question the use of this animal model to study the aqueous deficient dry eye.

The artificial tears containing *Artemia salina* also improved tear break-up time compared with the topical instillation of HPMC in the rabbits with induced dry eye (see Figure 1), which manifested the beneficial effect of the extract of *Artemia salina* on tear film stability. However, the comparison of POST measurements between both groups only showed an increase lower than 1 µL after the treatment with *Artemia salina* (see Table 1), not considered relevant for improving the severity of dry eye. To our knowledge, only the previous study of our research performed in healthy rabbits evaluated tear break-up time after a treatment containing dinucleotides, where there were no changes with the extract of 4% *Artemia salina* [30]. On the other hand, different studies demonstrated that the activation of the P2Y_2_ receptor by topical instillation of Up_4_U stimulates the production of mucins and lipids in the Goblet cells and Meibomian glands, respectively [15,16,34,35]. Considering that mucins and lipids are the responsible components for tear film stability [36], the possibility that the artificial tears with *Artemia salina* improves this stability in future clinical studies should not be discarded based on the hypothesis that the Gp4G present in the crustacean could act as an agonist of P2Y_2_ receptor.

Concerning the analysis of conjunctival cytologies by confocal microscopy, the secretagogue effect on the mucinous component of tears after the treatment with artificial tears containing *Artemia salina* was confirmed (see Figure 3). In terms of density of Goblet cells, the topical instillation of *Artemia salina* in the rabbits with induced dry eye did not show statistical differences with any group, including the healthy rabbits. This could indicate a protective effect against cell death induced by the instillation of 0.2% BAC. The results of the height of mucin cloud also demonstrated that the amount of mucin secreted by Goblet cells was higher after the treatment with *Artemia salina* due to there being no differences with the healthy rabbits and the mucin cloud increased compared with the untreated rabbits with dry eye. As mentioned above, the secretagogue effect on the mucinous component of tears is associated with the agonist action of dinucleotides on the P2Y_2_ receptors expressed in Goblet cells, despite only having been confirmed in the case of Up_4_U [15,34], but no other dinucleotides such as Ap_4_A or Gp_4_G. In this context, different studies performed on rabbits and rats found that topical instillation of Up_4_U stimulated the production of mucins in Goblet cells [15,34] and increased the concentration of MUC5A dissolved in tears [37,38]. In humans, Shigeyasu et al. [39,40] reported an increase in the concentration of sialic acid, a molecule that binds to the ends of mucin chains, in tears after the short- and long-term instillation of Up_4_U. 

Moreover, the treatment with artificial tears based on *Artemia salina* helped to protect the corneal epithelium against the damage produced by the topical instillation of 0.2% BAC due to corneal staining showed an improvement compared to the rabbits with induced dry eye treated and untreated with HPMC (see Figure 2). Again, the wound healing properties of the extract of *Artemia salina* were associated with the possible agonist action of its dinucleotides on P2Y_2_ receptors localized in the corneal epithelium as has been confirmed experimentally with Ap_4_A and Up_4_U [15,18,41,42,43]. Through this cellular mechanism, our research group found that the topical instillation of Ap4A accelerated wound healing by stimulating the cell migration of corneal epithelium [17,18]. Also, Fujihara et al. [15] demonstrated that the instillation of Up_4_U reduced corneal staining in an animal dry eye model. Conversely, other dinucleotides such as diadenosine triphosphate (Ap_3_A) and pentaphosphate (Ap_5_A) act as agonists of the P2Y_6_ receptor, which is also expressed in corneal epithelium, decelerating corneal wound healing by inhibiting cell migration [18,44,45].

Regarding the quantitative PCR of the conjunctival cytologies, the artificial tears based on *Artemia salina* reduced the mRNA levels of IL-1β and MMP9 compared with the instillation of HPMC (see Figure 5), suggesting a possibly beneficial effect on ocular surface inflammation. Nevertheless, the treatment with *Artemia salina* did not show statistical differences with the rabbits with induced dry eye used as positive controls. This lack of statistical significance was associated with the high variability that showed the positive controls, as is observed in the standard deviation of the results. The increase of sample size may have confirmed the trend of the extract of *Artemia salina* to reduce the mRNA levels of IL-1β and MMP9. Recent studies demonstrated the efficacy of Up4U or Diquafosol® to reduce ocular surface inflammation in both in vitro and in vivo dry eye models [38,46,47,48]. These studies found a reduction in the protein and mRNA expression of IL-1β and IL-6, among other biomarkers, which agrees with the results obtained with the artificial tears containing *Artemia salina* in the current study. Kim et al. [46] and Park et al. [48] found that this anti-inflammatory effect is mediated by the nuclear factor κB pathway, which also regulates the MMP9 expression [49]. However, it is unknown whether the anti-inflammatory properties of dinucleotides on the ocular surface are mediated by purinergic P2Y receptors, as occurs in other systemic pathologies [50], or are a consequence of the protective effect of these compounds against ocular surface damage and loss of homeostasis.

An important aspect of using the rabbit dry eye model induced by the topical instillation of BAC is that this model is not stable over time. Li et al. [51] reported that the signs of dry eye were sustained between 2 and 3 weeks after finishing the instillation of 0.1% BAC for 14 consecutive days. Under this context, different studies applied their treatments simultaneously to the instillation of BAC [52,53,54,55,56] or during the period of reversibility of the dry eye model [57,58,59,60,61]. In the current study, this aspect was considered for the final study design since it was previously confirmed that the instillation of the *Artemia salina* together with the 0.2% BAC offered better results than the instillation of *Artemia salina* during the period of reversibility.

Finally, the main limitation of the current study was that the interaction between the dinucleotides present in the extract of *Artemia salina* and purinergic P2 receptors localized on the ocular surface was not characterized. Therefore, it is only possible to hypothesize about the agonist action of Gp_4_G on the P2Y_2_ receptor to justify the beneficial effect of the artificial tears on tear secretion, corneal staining, the density of Goblet cells, and height of mucin cloud. On the other hand, the rabbit dry eye model induced by topical instillation of 0.2% BAC did not reproduce the chronic character of dry eye, this being the reason why it was not possible to evaluate the long-term treatment with the artificial tears containing *Artemia salina*.

## 4. Materials and Methods

### 4.1. Study Design

An experimental, prospective, and randomized study was carried out. All the trials were performed before (PRE) and after (POST) the topical instillation of the different treatments: saline solution as negative control (healthy group), 0.2% BAC + saline solution (dry eye group), 0.2% BAC + 0.24% hypromellose (dry eye + HPMC group), and 0.2% BAC + artificial tears containing *Artemia salina* (dry eye + *Artemia salina*).

The 0.2% BAC was used to induce the dry eye model by instilling 35 µL, twice per day (at 10:00 and 18:00), for 5 consecutive days, except on the last day when there was a single instillation during the morning [33]. For the 0.24% HPMC or artificial tears with *Artemia salina*, 35 µL were instilled 3 times per day (at 12:00, 14:00, and 16:00), for 5 consecutive days. In total, there were 9 instillations of 0.2% BAC or saline solution per eye in each rabbit and 15 instillations of HPMC, *Artemia salina*, or saline solution.

On the last day, the measurements were taken 20 min after the last instillation at 16:00, when dinucleotides produce the maximum effect on tear secretion [13,14]. The order of the trials was as follows: tear secretion, slit-lamp examination (including tear break-up time), and conjunctival cytology.

### 4.2. Animals

A total of 20 male New Zealand white rabbits were used in the study, including both eyes in the same experimental group (n_eyes_ = 40). The rabbits were randomly divided into four groups: 5 rabbits as negative control (healthy group, n_eyes_ = 10), 5 rabbits with induced dry eye as a positive control (dry eye group, n_eyes_ = 10), 5 rabbits with induced dry eye and treated with HPMC (dry eye + HPMC group, n_eyes_ = 10), and 5 rabbits with induced dry eye and treated with artificial tears of *Artemia salina* (dry eye + *Artemia salina*, n_eyes_ = 10).

The rabbits were provided by the animal facility of the Faculty of Veterinary of the Complutense University of Madrid. They were kept in cages for 7 days before experimentation to get them used to their new housing conditions. Their weight was between 3.0 and 3.5 kg and they had free access to food and water. The rabbits were under controlled conditions: 12 h light–dark cycles, a temperature of 18 °C, and a humidity of 30%.

### 4.3. Artificial Tears

The artificial tears based on an extract of *Artemia salina* were manufactured and provided by the company Avizor (Avizor, Madrid, Spain). The qualitative composition of these artificial tears was as follows: an extract of 4% *Artemia salina* containing 10 µM of Gp_4_G as an active ingredient, 0.24% HPMC as a thickening agent, boric acid and borax as a buffer, and CaCl_2_, KCl, and MgCl_2_ as electrolytes. The artificial tears had an osmolality of 248 mOsm/kg, a pH of 7.1, and a viscosity of 5.6 cP.

The treatment with HPMC had the same composition as the artificial tears of *Artemia salina* but with no crustacean extract. The saline solution used during the experiments was also provided by Avizor, while the BAC was provided by Merck (Merck, Darmstadt, Germany).

### 4.4. Tear Secretion and Tear Break-Up Time

Tear secretion with anesthesia was measured by performing a Schirmer’s test (Aiesi; Naples, Italy) for 5 min. The paper strip was positioned in the inferior lid and the rabbit’s eyes were closed to avoid the reflex secretion associated with blinking. Each millimeter of the paper strip soaked corresponded to 1 µL of tear secretion. The topical anesthesia was induced by the instillation (2 drops in 5 min) of commercial eye drops containing 4 mg/mL of oxybuprocaine hydrochloride and 1 mg/mL of tetracaine hydrochloride (Alcon Cusí, Barcelona, Spain). Tear secretion was measured 5 min after the last instillation of anesthesia.

Tear break-up time was measured during the slit-lamp examination. It was evaluated after the instillation of 2 µL of commercial 2% fluorescein sodium (Alcon Cusí) over the ocular surface. Three consecutive measurements were taken with a timer after manually forcing the rabbits to blink.

### 4.5. Slit-Lamp Examination

The signs of ocular surface damage were examined with a VX75 slit lamp (Luneau Technology, Chartres, France). The severity of corneal staining and conjunctival hyperemia was quantified by using the Efron Grading Scales [62], which classify this severity as follows: normal (0), trace (1), mild (2), moderate (3), and severe (4). Corneal staining was measured immediately after measuring tear break-up time with fluorescein sodium.

### 4.6. Conjunctival Cytology

The medical device EYEPRIM (Opia Technologies, Paris, France) was used to collect the superficial conjunctival cells. Two cytologies of both superior and inferior quadrants of the bulbar conjunctiva were taken. The superior cytology was used to quantify the density of Goblet cells and the height of mucid cloud of these cells, while the inferior cytology was used to quantify the mRNA levels of interleukin 1β (IL-1β), IL-6, and metalloproteinase 9 (MMP9) by polymerase chain reaction (PCR).

The superior cytology was fixed in 96% ethanol at 4 °C for 24 h to be stained with the hematoxylin-periodic acid Schiff procedure posteriorly. For the visualization of the Goblet cells, a confocal microscopy system FV1200 (Olympus, Tokyo, Japan) was used, while the images were analyzed with the ImageJ software (National Institutes of Health; Bethesda, MD, USA). The samples were excited by a wavelength of 559 nm and the light emission was filtered for a range between 580 and 620 nm. Magnifications of x20 and x40 were used for the quantification of the density of Goblet cells and the height of mucin cloud, respectively. The Z-stacking to visualize the three-dimensional cells was performed with a pupil diameter of 180 µm and a stack interval of 0.25 µm. All of these procedures were previously detailed by Peral and Pintor [63].

The density of Goblet cells was quantified in 5 different regions of each sample, while the height of mucin cloud, including the cell thickness, was quantified in 15 different cells.

### 4.7. Quantitative PCR

The inferior conjunctival cytology was fixed in RNAlater (Thermo Fisher Scientific, Waltham, MA, USA) at 4 °C for 24 h. Then, RNAlater was removed and the samples were stored at −80 °C until being processed.

The RNA isolation and purification of the samples were performed with the commercial kits QIAshredder and RNeasy Mini Kit (Qiagen, Hilden, Germany), following the manufacturer’s instructions.

Twenty-two µL of the total RNA were used for the first-strand cDNA synthesis that was performed with the High Capability cDNA Reverse Transcription Kit and random hexamer primers (Thermo Fisher Scientific). The quantitative PCR was carried with the QuantStudio 3 system (Thermo Fisher Scientific) by using the cDNA, Quantitect SYBR Green Kit (Qiagen), and specific primers of IL-1β, IL-6, and MMP9 (Table 2). The hypoxanthine-guanine phosphoribosyltransferase 1 (HPRT1) gene was used as an internal control to normalize the mRNA relative expression. Each sample was triplicated and negative controls were included in all the measurements. The thermal cycler program was as follows: 15 min at 95 °C, 40 cycles of 15 s at 94 °C, 30 s at 55 °C, and 34 s at 72 °C.

The analysis of the melting curves confirmed the specificity of the primers and the absence of primer-dimers. Finally, both the stability of the HPRT1 gene and the analysis of the quantitative PCR data were performed by the 2^−ΔCt^ method.

### 4.8. Statistical Analysis

The statistical analysis was performed with the SPSS 23 software (IBM; Chicago, IL, USA). The normality of all the variables was checked using the Shapiro–Wilk test. The comparison between the measurements before (PRE) and after (POST) the instillation of the different treatments was carried out with the Student’s *t*-test for paired samples (normal distributions) or the Wilcoxon signed-rank test (non-normal distributions). Besides, the comparison of the effect of the different treatments (difference between PRE and POST measurements) between groups was performed using the Student’s *t*-test for independent samples (normal distributions) or the Mann–Whitney U test (non-normal distributions). A statistical significance of 95% (*p* < 0.05) was established in all the tests.

The analyzed variables were: tear secretion, tear break-up time, corneal staining, conjunctival hyperemia, density of Goblet cells, height of mucin cloud, and mRNA levels of IL-1β, IL-6, and MMP9. Results are reported as mean ± standard deviation (SD).

## 5. Conclusions

The artificial tears based on an extract of 4% *Artemia salina* showed secretagogue properties on aqueous and mucinous components of tears, accompanied by a protective effect against ocular surface damage and inflammation in a rabbit dry eye model. Thus, the potential of these artificial tears as a secretagogue agent for dry eye treatment was confirmed, which opens the door for future clinical trials and studies to extrapolate the findings to dry eye patients.

## 6. Patents

Pintor Just, J.J.; Pérez de Lara, M.J.; Huete Toral, F.; Colligris, B.; Carracedo Rodríguez, J.G. WO/2018/015582—Preparation and use of an extract of *Artemia salina* to treat the ocular surface. 2017.

## Figures and Tables

**Table 1:** Values of all the variables under study before (PRE) and after (POST) the instillation of the different treatments for 5 consecutive days. The statistical comparison was performed between the values PRE and POST in each group.

Variable	Group (*n* = 5, Each One)	Mean ± SD		*p*-Value
		PRE	POST	
Tear secretion (μL)	Healthy	7.0 ± 3.4	6.0 ± 3.1	0.373
	Dry eye	7.8 ± 2.2	6.3 ± 2.3	0.081
	Dry eye + HPMC	8.1 ± 2.5	7.4 ± 3.0	0.477
	Dry eye + Artemia	7.3 ± 2.7	12.0 ± 3.4	0.008 ^
Tear break-up time (s)	Healthy	4.2 ± 0.7	4.1 ± 1.3	0.896
	Dry eye	5.4 ± 2.7	1.7 ± 0.6	0.005 *
	Dry eye + HPMC	5.2 ± 1.5	1.7 ± 0.9	0.005 ^
	Dry eye + Artemia	4.1 ± 1.1	2.5 ± 1.0	<0.001 *
Corneal staining (score)	Healthy	1.90 ± 0.88	1.70 ± 1.06	0.516
	Dry eye	1.60 ± 0.84	4.00 ± 0.00	0.004 *
	Dry eye + HPMC	1.70 ± 0.95	3.70 ± 0.48	0.007 *
	Dry eye + Artemia	2.30 ± 0.67	3.00 ± 0.94	0.140
Conjunctival hyperemia (score)	Healthy	0.00 ± 0.00	0.10 ± 0.32	0.317
	Dry eye	0.00 ± 0.00	3.40 ± 0.70	0.004 *
	Dry eye + HPMC	0.00 ± 0.00	3.50 ± 0.85	0.004 *
	Dry eye + Artemia	0.00 ± 0.00	2.90 ± 0.57	0.004 *
Density of Goblet cells (cells/mm^2^)	Healthy	641.2 ± 216.7	646.5 ± 263.5	0.960
	Dry eye	911.7 ± 319.9	291.5 ± 103.2	<0.001 *
	Dry eye + HPMC	850.8 ± 297.1	382.9 ± 336.2	0.022 *
	Dry eye + Artemia	851.8 ± 248.4	578.4 ± 254.1	0.071
Height of mucin cloud (μm)	Healthy	18.4 ± 1.0	18.1 ± 1.6	0.532
	Dry eye	16.8 ± 2.1	10.8 ± 1.7	<0.001 *
	Dry eye + HPMC	16.7 ± 1.2	14.0 ± 4.0	0.053
	Dry eye + Artemia	16.6 ± 1.9	15.5 ± 1.9	0.139
mRNA levels of IL-1β (fold change)	Healthy	6.430 ± 8.572	3.466 ± 3.067	0.878
	Dry eye	7.758 ± 11.702	42.369 ± 42.889	0.009 ^
	Dry eye + HPMC	4.103 ± 2.772	46.116 ± 26.483	0.001 *
	Dry eye + Artemia	1.295 ± 1.838	12.408 ± 10.426	0.012 ^
mRNA levels of IL-6 (fold change)	Healthy	0.072 ± 0.082	0.416 ± 0.457	0.028 ^
	Dry eye	0.046 ± 0.036	0.338 ± 0.401	0.017 ^
	Dry eye + HPMC	0.051 ± 0.086	0.230 ± 0.195	0.009 ^
	Dry eye + Artemia	0.026 ± 0.033	0.175 ± 0.132	0.025 ^
mRNA levels of MMP9 (fold change)	Healthy	0.028 ± 0.034	-	-
	Dry eye	0.042 ± 0.059	0.314 ± 0.266	0.009 ^
	Dry eye + HPMC	0.030 ± 0.017	0.525 ± 0.483	0.005 ^
	Dry eye + Artemia	0.012 ± 0.017	0.174 ± 0.176	0.012 ^

* *p* < 0.05, Student’s *t*-test for paired samples (normal distributions); ^ *p* < 0.05, Wilcoxon signed-rank test (non-normal distributions); Artemia: *Artemia salina*; HPMC: hypromellose.

**Table 2:** Sequences of the different primers analyzed by quantitative polymerase chain reaction.

Primer	Sequence (Forward/Reverse)
HPRT1	5′-CTGGCAAAACAATGCAGACCT-3′/ 5′-GTCCTTTTCACCAGCAGGCTT-3′
IL-1β	5′-TTGAAGAAGAACCCGTCCTCTG-3′/ 5′-CTCATACGTGCCAGACAACACC-3′
IL-6	5′-GCCTCACAAACTTCCTGGAG-3′/ 5′-GATGGTGTGTTCTGACCGTG-3′
MMP9	5′-AAGACGCAGACGGTGGATTC-3′/ 5′-ACTCACACGCCAGAAGAAGC-3′

HPRT1: hypoxanthine-guanine phosphoribosyltransferase 1; IL-1β: interleukin 1β; IL-6: interleukin 6; MMP9: metalloproteinase 9.

## Data Availability

The data used to support the findings of this study are available from the corresponding author upon request.
